# Scalable Differentiation of Human iPSCs in a Multicellular Spheroid-based 3D Culture into Hepatocyte-like Cells through Direct Wnt/β-catenin Pathway Inhibition

**DOI:** 10.1038/srep32888

**Published:** 2016-09-12

**Authors:** Giuseppe Pettinato, Rajesh Ramanathan, Robert A Fisher, Martin J. Mangino, Ning Zhang, Xuejun Wen

**Affiliations:** 1 Department of Chemical and Life Science Engineering, Virginia Commonwealth University, Richmond, VA, USA; 2 Department of Surgery, Beth Israel Deaconess Medical Center, Harvard Medical School, Boston, MA, USA; 3 Department of Biomedical Engineering, Virginia Commonwealth University, Richmond, VA, USA; 4 Department of Surgery, Virginia Commonwealth University Medical Center, Richmond, VA, USA; 5 Shanghai East Hospital, The Institute for Biomedical Engineering and Nano Science, Tongji University School of Medicine, Shanghai 200120, People’s Republic of China

## Abstract

Treatment of acute liver failure by cell transplantation is hindered by a shortage of human hepatocytes. Current protocols for hepatic differentiation of human induced pluripotent stem cells (hiPSCs) result in low yields, cellular heterogeneity, and limited scalability. In the present study, we have developed a novel multicellular spheroid-based hepatic differentiation protocol starting from embryoid bodies of hiPSCs (hiPSC-EBs) for robust mass production of human hepatocyte-like cells (HLCs) using two novel inhibitors of the Wnt pathway. The resultant hiPSC-EB-HLCs expressed liver-specific genes, secreted hepatic proteins such as Albumin, Alpha Fetoprotein, and Fibrinogen, metabolized ammonia, and displayed cytochrome P450 activities and functional activities typical of mature primary hepatocytes, such as LDL storage and uptake, ICG uptake and release, and glycogen storage. Cell transplantation of hiPSC-EB-HLC in a rat model of acute liver failure significantly prolonged the mean survival time and resolved the liver injury when compared to the no-transplantation control animals. The transplanted hiPSC-EB-HLCs secreted human albumin into the host plasma throughout the examination period (2 weeks). Transplantation successfully bridged the animals through the critical period for survival after acute liver failure, providing promising clues of integration and full *in vivo* functionality of these cells after treatment with WIF-1 and DKK-1.

Liver dysfunction that is caused by cirrhosis, hepatitis, or acute liver failure is frequently fatal. To date, the most effective therapy for acute liver failure is liver transplantation. However, donor liver shortages and the requirement for lifelong immunosuppression have limited the use of liver transplantation^1^,^2^,^3^,^4^,^5^. As a result, hepatocyte transplantation and bioartificial liver (BAL) devices containing active hepatocytes that remove toxins and supply key physiological active molecules to sustain hepatic function have been successfully used to bridge patients to native regeneration or organ transplantation^6^. These therapeutic modalities, however, are limited by the lack of human livers as a source of hepatocytes and limitations of xenogenic sources. Additionally, practical limitations of hepatocyte-based therapies include the rapid deterioration in function of primary hepatocytes in culture, and their variable viability upon recovery from cryopreservation^7^,^8^,^9^.

Human induced pluripotent stem cells (hiPSCs) hold great promise in personalized regenerative medicine due to their pluripotent potential, high proliferative index, and absence of rejection and ethical controversy. iPSC can be generated by retro-engineering adult differentiated cells back into a pluripotent state through the addition of various stemness factors^10^,^11^,^12^,^13^,^14^. hiPSCs demonstrate three-germ layer differentiation potential and can be differentiated into a wide variety of cell types, including hepatocyte-like cells (HLCs)^15^,^16^,^17^. HLCs that are derived from hiPSCs represent a promising, potentially inexhaustible alternative source of hepatocytes in cell therapy and bioengineered livers for the treatment of hepatic diseases^18^, pharmaceutical testing^19^, as well as the study of the developmental biology of hepatogenesis^20^,^21^. Theoretically, hiPSC-derived hepatocytes have the potential to enable autologous cell transplantation and thereby mitigate the adverse effects of immune sensitization and rejection^18^. The translational potential of stem cell-derived HLCs has not been fully demonstrated due to the large cell doses required per transplantation. Current differentiation protocols for generating HLCs from hiPSCs are limited by low yields and cellular heterogeneity.

An increasing number of studies have investigated hepatic differentiation of hESCs or hiPSCs and have provided insights into differentiation strategies. These studies have, in general, reached the consensus that the differentiation yields and culture uniformity are subject to the effects of multiple variables in the culture, including the form of the hiPSCs to start with, the differentiation substrates, the induction schemes, and scalability of the protocol. Hepatic differentiation of hESCs or hiPSCs usually starts by one of three methods, i.e., embryoid bodies (EBs) that are subsequently plated on diverse substrates^24^,^25^, differentiation on mouse embryonic fibroblasts feeder layers^26^,^27^, or differentiation on adherent feeder-free cultures^28^. EBs are 3-dimensional (3-D) hiPSC cell aggregates that can differentiate into cells of all three germ layers (endoderm, ectoderm, and mesoderm)^29^. Events in the *in vitro* lineage-specific differentiation process within the EBs recapitulate those seen *in vivo* in the developing embryo^30^, which justifies the use of EBs as a model to simulate the *in vivo* differentiation of hPSCs under *in vitro* culture conditions^31^. Differentiation protocols starting from EBs are more scalable due to their higher tolerated density of cells within the clusters and the ability to be maintained in a suspension culture. Previously described techniques to reproducibly generate embryoid bodies from hiPSCs or hESCs have used the xeno-factor, rho-associated kinase inhibitors (ROCKi), and/or centrifugation^32^. Recently, robust scalable production of homogeneous and synchronous hEBs from singularized hPSCs using non-adhesive round-bottom hydrophilic microwell arrays and eliminating both ROCKi xeno-factor and/or centrifugation has been demonstrated by our group^29^,^33^. This new technique has allowed us to produce hiPSC-derived synchronized hEBs in large quantities for direct differentiation into the desired cell lineages.

Embryonic liver development follows three phases characterized by the formation of the definitive endoderm (DE), hepatoblast expansion and proliferation, and differentiation of hepatoblasts into mature, functional hepatocytes. Hepatoblasts are bipotential stem cells capable of giving rise to both major lineages of the liver: hepatocytes and biliary epithelial cells (cholangiocytes)^34^. The Wnt and β-catenin demonstrate individual as well as junctional effects in controlling postnatal liver development^22^. Increased β-catenin translocation to the nucleus correlates with an increase in cell proliferation^23^, whereas the Wnt pathway is considered as the major regulator of polarity and cell fate specifications^35^. The effect of the Wnt and β-catenin on liver embryogenesis follows a highly temporally regulated profile^36^,^37^. When combined, the Wnt/β-catenin pathway plays an important role in the hepato-biliary differentiation toward hepatocytes^38^,^39^, whereas stabilization of the β-catenin alone leads to increased propensity toward cholangiocytes over hepatocytes^40^. Through the Wnt/β-catenin inhibition, it is possible to promote progression to hepatocytes at the hepato-biliary differentiation stage. During the phase II of liver development, hepatoblasts or hepatic progenitors undergo expansion while maintaining their de-differentiated state. Commitment to a hepatic fate is regulated by an array of the liver-enriched transcriptional factors that are present during phase III^41^,^42^. Current conventional differentiation protocols follow a stepwise process from the initial endoderm formation, passing through hepatic progenitor cell induction, toward a mature hepatic phenotype without taking into account the important role of Wnt/β-catenin inhibition. The soluble factors that are administered at different stages of differentiation include: Activin A for the endoderm formation, FGF family factors for the progenitor hepatic specification, with the addition of BMP4 in some cases, and finally Oncostatin M and HGF for the maturation step^16^,^43^,^44^,^45^,^46^,^47^. Notable limitations with current protocols include low scalability, remnant immature genotypes after differentiation, and poor long-term cell functionality following transplantation^48^.

In this study, we have developed a novel 3D multicellular spheroid culture-based hepatic differentiation protocol that starts from hEBs and employs two inhibitors of the Wnt/β-catenin pathway to mimic the differentiation stage during hepatogenesis *in vivo*. The scalability of our *in vitro* hepatic differentiation protocol allows the production of human hepatocytes in large quantities for transplantation therapy. The functionality of hiPSC-derived HLCs was investigated in an animal model of acute liver failure.

## Results

### Differentiation of hiPSC embryoid bodies (hiPSC-EBs) in 3D culture into hepatocyte-like cells (HLCs)

**Figure 1:**
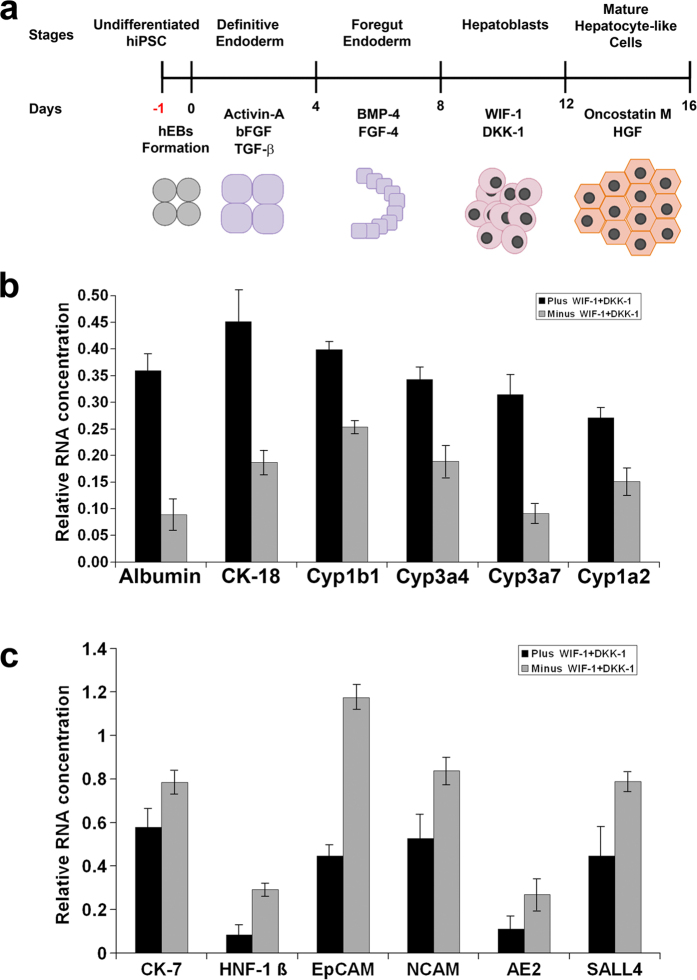
**(a)** Schematic representation of the 4-stage differentiation protocol and the major regulatory factors administered at each stage: the differentiation protocol recapitulates the stages of ontogenic development of liver; starting from the undifferentiated human induced pluripotent stem cells (hiPSCs), the cells undergo differentiation to Definitive Endoderm (DE), followed by Foregut Endoderm (FE) from where the Hepatic Progenitor Cells (HPCs) or Hepatoblasts arise. The final maturation step leads to mature Hepatocyte-Like Cells (HLCs). (**b**) The hiPSC-EBs differentiated with both WIF-1 and DKK-1 exhibited greater expressions of hepatocyte-specific markers relative to the ones differentiated without the WIF-1 and DKK-1. Data presented as mean ± SD (n = 3). (**c**) The hiPSC-EBs differentiated without WIF-1 and DKK-1 showed greater expressions of cholangiocyte-specific markers relative to the ones differentiated with both WIF-1 and DKK-1. Data presented as mean ± SD (n = 3).

Embryoid bodies were produced reliably and efficiently with high viability using agarose micro-well arrays and Teflon stamps^29^. An 80% confluent six-well plate containing 1.2 × 10^6^ dissociated hiPSC produced approximately 280 embryoid bodies (Supplementary Fig. 1). The hiPSC-EBs underwent a 4-stage hepatic differentiation process in a continuous 3D culture. The differentiation protocol recapitulates the developmental stages that occur during embryogenesis *in vivo* (Fig. 1a). Starting from pluripotent stem cells (PS), the four stages were definitive endoderm (DE), foregut endoderm (FE), hepatic progenitor cells or hepatoblast (HPC) and mature hepatocytes (MH). Each stage of the differentiation protocol lasted 4 days with two every-other-day medium changes. The protocol used two novel factors for Wnt inhibition: Wnt inhibitory factor 1 (WIF-1) and Dickkopf-1 (DKK-1)^49^ at the HPC stage. The working concentrations of WIF-1 and DKK-1 followed the suggested manufacturer ranges and literature review of studies in mouse^50^,^51^ and human cell lines^52^,^53^. The concentrations that showed the maximum effect in WNT inhibition in those studies were used.

To evaluate the effect of the two Wnt inhibitors on hepatic differentiation and ensure the reproducibility of our results, all the experiments were performed in parallel starting from the same batch of hiPSC-EBs for both the treated and non-treated control (with and without inhibitors), as well as the undifferentiated hiPSC-EBs and adult human hepatocyte (negative and positive controls).

At the end of the differentiation process, we used semi-quantitative PCR to analyze markers for cholangiocyte- and hepatocyte-specific gene expression. The presence of the two Wnt inhibitors in the differentiation protocol resulted in increased propensity of the differentiating hiPSC-EBs toward the two different lineages. The hiPSC-EBs differentiated with both WIF-1 and DKK-1 exhibited much higher expressions of hepatocyte-specific markers relative to the ones differentiated without the two Wnt inhibitors (Fig. 1b). The hiPSC-EBs differentiated without WIF-1 and DKK-1 demonstrated greater expressions of cholangiocyte-specific markers (Fig. 1c). The differences between the conditions with and without WIF-1 and DKK-1 in gene expression were all statistically significant (p < 0.0001).

**Figure 2:**
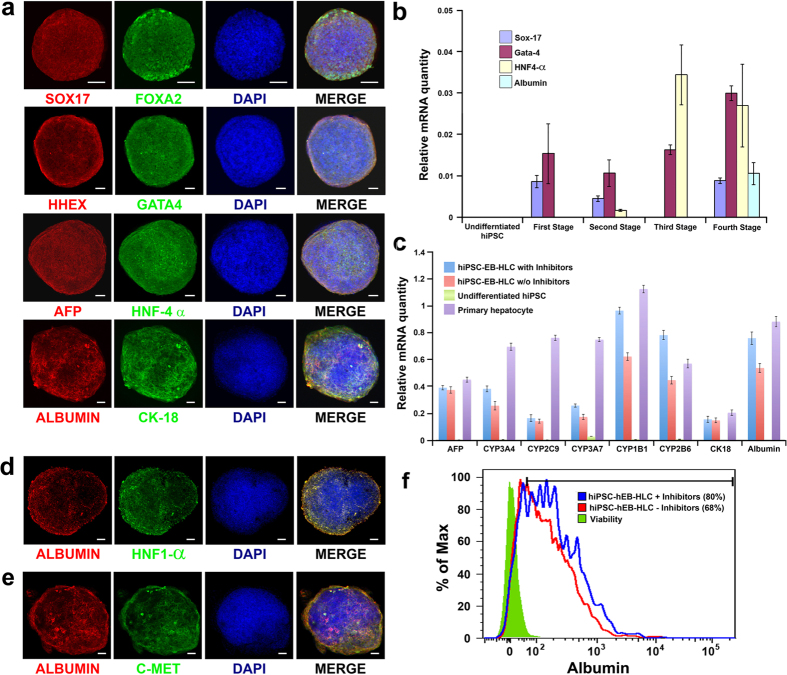
(**a**) Stage-specific protein expressions of hiPSC-EBs during the differentiation process. SOX17 and FOXA2 for the definitive endoderm stage; HHEX and GATA4 for the foregut endoderm; AFP and HNF-4α for the hepatic progenitor cells; ALBUMIN and CK-18 for the mature HLCs. DAPI stains for cell nuclei. Scale bar 100 μm. (**b**) Stage-specific gene expression analysis by Real-Time PCR. The relative quantities of stage-specific genes were measured at the mRNA level to follow the progression of the differentiation process. Sox17 as the definitive endoderm marker; Gata4 as the foregut endoderm marker; HNF-4α as the hepatic progenitor cells marker; Albumin was used to determine the final maturation for the hepatocyte-like cells (HLCs). Undifferentiated cells were used as negative control. (**c**) quantitative RT-PCR displayed the presence of mRNA for AFP, five P450 isoforms (Cyp3A4, Cyp2C9, Cyp3A7, Cyp1B1, and Cyp2B6), Albumin, and CK18 in the terminally differentiated hiPSC-EB-HLCs with and without inhibitors. Gene expression for the condition with inhibitors was greater compared with the one without inhibitors for any gene tested; (**d,e**) Following the differentiation program, terminally differentiated hiPSC-EB-HLCs expressed mature hepatocyte-specific markers, as evidenced by co-staining of ALBUMIN and HNF-1α, and ALBUMIN and C-MET. Scale bar 100 μm. (**f**) FACS analysis for albumin positive cells showed a higher percentage of albumin producing cells in the condition with inhibitors compared with the one without inhibitors (80% vs 68%).

To ensure the stepwise differentiation of the hiPSC-EBs using our protocol, stage-specific protein analyses were performed at the end of individual stages. The differentiating hiPSC-EBs exhibited a temporal regulated pattern of stage-specific intracellular hepatic protein expression at the end of each individual stage, including FOXA2 and SOX17 at the end of definitive endoderm^16^,^44^,^54^, HHEX^55^,^56^,^57^ and GATA-4^58^,^59^ at the end of foregut endoderm, HNF-4α and AFP at the end of hepatic endoderm^60^,^61^,^62^,^63^, and Albumin and CK-18 at the end of mature hepatocyte stage^61^,^62^,^63^ (Fig. 2a).

To determine the gene expression profile of the differentiating hiPSC-EBs, we used quantitative RT-PCR (qRT-PCR) at various time points during the differentiation protocol to measure the relative quantities of stage-specific genes at the mRNA level (Fig. 2b). Undifferentiated hiPSCs were used as negative controls. The mRNA of the undifferentiated hiPSCs was negative for markers of all four differentiation stages. In general, expressions of stage-specific genes by differentiating hiPSC-EBs peaked at the respective stages and gradually declined subsequent to that stage. The only exception was GATA-4, a marker for stage II (foregut endoderm), which was induced as early as stage I and peaked at stage IV. At stage IV, albumin mRNA expression was seen. Quantitative RT-PCR for both conditions, with and without WIF-1 and DKK-1, displayed the presence of mRNA for five P450 isoforms (Cyp1B1, Cyp2C9, Cyp3A4, Cyp2B6 and Cyp3A7), Alpha fetoprotein, Albumin, and CK18 in the terminally differentiated hiPSC-EB-HLCs (Fig. 2c). In particular, hiPSC-EBs treated with the protocol containing WIF-1 and DKK-1 showed a higher expression pattern for all the markers compared to the hiPSC-EBs treated with the protocol without the two inhibitors. Undifferentiated hiPSCs were used as negative control and human primary hepatocytes were used as positive control. Following the differentiation program, terminally differentiated hiPSC-EB-HLCs expressed a repertoire of mature hepatocyte-specific proteins, as evidenced by immunohistochemical co-staining of ALBUMIN and HNF-1α, and ALBUMIN and C-MET^61^,^62^,^63^ (Fig. 2d–e). The comparison for the hepatic differentiation yield between the two protocols with and without WIF-1 and DKK-1 was evaluated by FACS analysis using the albumin-positive cells as the reference marker. The results showed an increased yield of Albumin positive cells in the hiPSC-EBs treated with the protocol with the two inhibitors compared with the one without inhibitors (80% vs 68% respectively) (Fig. 2f). A confirmation of this result for the protocol with both inhibitors was obtained by counting under confocal microscope the fraction of albumin-positive cells in each optical section averaged over a minimum of 10 microscopic fields for each cluster with a minimum of 50 different clusters per differentiation condition. Our differentiation protocols with both Wnt inhibitors WIF-1 and DKK-1 had consistently yielded an over 80% high-purity hepatocytes population when compared to a 70% hepatic differentiation yield that is usually seen with other differentiation protocols^54^. Supplementary Fig. 2 shows representative images used for cell counting to determine the hepatic differentiation yield.

### hiPSC-EB-HLCs displayed morphology and *in vitro* functional hepatic characteristics

Morphological assessment of hiPSC-EBs undergoing differentiation in 3D culture revealed a progressive increase in cluster size from approximately 500 μm to 800–1,000 μm by the end of differentiation without any core necrosis at any time (Supplementary Fig. 3). In order to further assess the cellular morphology at the end of the differentiation protocol, the hiPSC-EB-HLCs in 3D culture were placed in a Matrigel-coated plate and allowed to adhere. Over the course of 1 week, the hiPSC-EB-HLCs adhered to the surface of the plate and began to spread in a monolayer. Light microscopy showed that hiPSC-EB-HLCs were morphologically polygonal with enriched cytoplasmic granules (Supplementary Fig. 4), replicating the morphological features of polygonal, vacuolated primary human hepatocytes^28^. This result was observed for both conditions studied.

**Figure 3:**
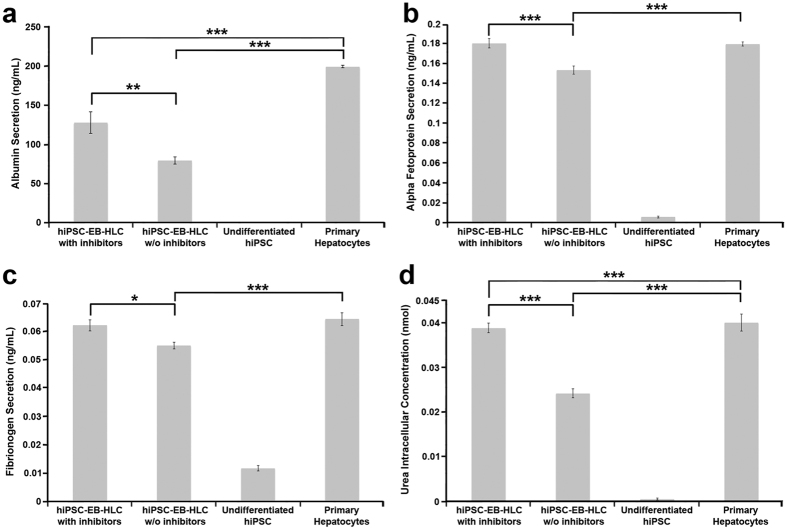
Secretion of hepatic proteins by hiPSC-EB-HLCs. The conditioned medium from hiPSC-EB-HLCs was collected 48 hours following the completion of the differentiation process for both conditions with and without inhibitors. (**a**) Albumin, (**b**) Alpha Fetoprotein (AFP) and (**c**) fibrinogen were detected in the medium and (**d**) intracellular Urea was detected. The difference in secretion between the conditions with inhibitors was statistically significant with respect to the condition without inhibitors. Undifferentiated hiPSCs were used as negative control. The results are representative of at least three independent experiments. Data presented as mean ± SD (n = 3). *p < 0.05; **p < 0.01; ***p < 0.001.

Examination of the conditioned culture medium indicated secretion of hepatic proteins by the hiPSC-EB-HLCs 48 hours following the completion of the differentiation process with our protocol with and without WIF-1 and DKK-1. hiPSC-EB-HLCs showed a different secretion pattern for all the proteins examined between the conditions with and without inhibitors, with increased secretion with the inhibitors. In particular, the condition with the inhibitors demonstrated increased albumin secretion (120–130 ng/ml vs. 76–80 ng/ml for 5 × 10^5^ cells respectively; p = 0.0046). This corresponded approximately to 60% and 40% respectively of albumin production by primary human hepatocytes (128 ng/ml vs. 199 ng/ml, p < 0.0009; 80 ng/ml vs. 199 ng/ml, p < 0.0001). The condition with the two inhibitors showed statistically significantly higher secretion of both alpha fetoprotein (AFP) (0.18 ng/ml vs. 0.15 ng/ml, p = 0.0007), and fibrinogen (0.062 ng/ml vs. 0.055 ng/ml, p = 0.0175) relative to the condition without the inhibitors. Both AFP and fibrinogen in the conditions with WIF-1 and DKK-1 showed total protein concentration at levels that were equivalent to those of primary human hepatocytes (AFP: 0.18 ng/ml vs. 0.19 ng/ml, p = 0.69; Fibrinogen: 0.062 vs. 0.064, p = 0.0015) (Fig. 3a–c). In addition, the hiPSC-EB-HLCs under the condition with the inhibitors demonstrated an intracellular urea concentration that was statistically significantly higher relative to that of the hiPSC-EB-HLCs under the condition without the inhibitors (0.0388 nmol vs. 0.024 nmol, p < 0.0001) (Fig. 3d). Undifferentiated hiPSCs were used as negative control, in which production of the proteins was absent at all times (p < 0.01) (Fig. 3).

**Figure 4:**
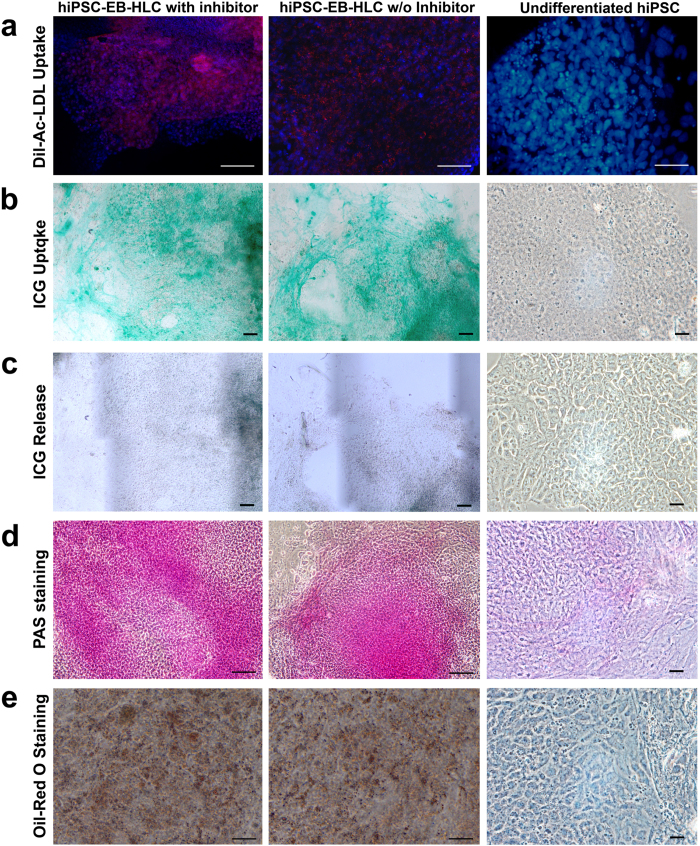
The resultant hiPSC-EB-HLCs displayed functional activities typical of mature primary hepatocytes, such as (**a**) Acetylated low-density lipoprotein (DiI-ac-LDL) uptake in red; (**b**) Indocyanine green (ICG - Cardiogreen) uptake; (**c**) ICG release after 6 hours; (**d**) glycogen storage indicated by PAS staining; and (**e)** cytoplasmic accumulation of neutral triglycerides and lipids indicated by Oil-Red O staining for both conditions with and without inhibitors. Undifferentiated hiPSCs were used as negative control. Scale bar 100 μm.

To assess the functional activities of the hiPSC-EB-HLCs, differentiated hiPSC-EB-HLCs in 3D culture were placed in a Matrigel-coated plate and allowed to adhere and spread in a monolayer (Supplementary Fig. 5). The hiPSC-EB-HLCs of both conditions with and without WIF-1 and DKK-1 displayed similar functional activities typical of mature primary hepatocytes^64^,^65^, such as acetylated low-density lipoprotein (DiI-ac-LDL) uptake (Fig. 4a), indocyanine green (ICG - Cardiogreen) absorption and release after 6 hours (Fig. 4b,c). glycogen storage (Fig. 4d), and cytoplasmic accumulation of neutral triglycerides and lipids (Fig. 4e). Undifferentiated hiPSCs were used as negative control (right panel of Fig. 4) and did not demonstrate any of the activities above.

**Figure 5:**
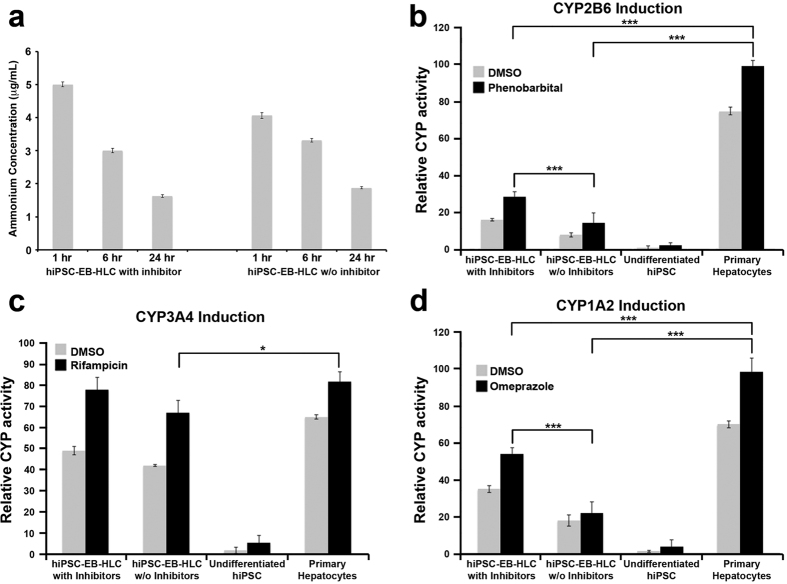
(**a**) Ammonium metabolism assay over a 24-hour period for both conditions with and without inhibitors; Cytochrome P450 (CYP450) induction analysis comparing the two experimental conditions with and without inhibitors. Several CYP enzymes were evaluated through incubation of the cells with different inducers: (**b**) Phenobarbital for the CYP2B6, (**c**) Rifampicin for the CYP3A4 and (**d**) Omeprazole for the CYP1A2 for a period of 72 hours. DMSO was used as control to test the basal activity of different CYP450. Data presented as mean ± SD (n = 3). *p < 0.05; ***p < 0.001.

Ammonia metabolism via the urea cycle is an essential function of hepatocytes. Ammonia metabolism was evaluated by changes in ammonium concentration in the cell culture supernatant for both experimental conditions over a 24-hour period after addition of ammonium chloride of known concentration. Ammonium chloride standard of 1 mM was added to culture dishes containing 100 differentiated hEBs in suspension deriving from hiPSC differentiated with the protocol with and without WIF-1 and DKK-1. Supernatant was collected and ammonium concentration was measured at 1-, 6- and 24-hour intervals after ammonium chloride addition. There was a steady decrease in ammonium concentration in the supernatant over a 24-hour period for both conditions (Fig. 5a). In particular, there was not a statistically significant decrease in ammonium concentration between the sample treated with the two inhibitors and the one without. However, the levels of ammonium chloride at 24 hours showed a higher percentage of loaded ammonium that was metabolized by the hiPSC-EB-HLCs with the two inhibitors compared to the cells treated without inhibitors (70.15 ± 5.12% vs. 60.32 ± 3.25% respectively).

Next we examined the detoxification abilities of the hiPSC-EB-HLCs *in vitro* by characterizing the activities of Cytochrome P450 (CYP450) enzymes, the major hepatic enzymes that perform detoxification. Three CYP isoforms were tested by measuring the increase in CYP isoform gene expression in response to exposure to their respective inducers for 72 hours. The three inducers and CYP isoforms were Omeprazole (CYP1A2), Phenobarbital (CYP2B6), and Rifampicin (CYP3A4). DMSO was used as a control in cell co-culture to test the basal activity of the different CYP450. Our results indicated significant increases in the activities of all the tested isoforms of CYP450 in cell culture relative to the DMSO control (Fig. 5b–d). The hiPSC-EB-HLCs treated with the protocol containing WIF-1 and DKK-1 displayed increased CPY expression when compared to the one without inhibitors, in response to Phenobarbital (28.16 ± 2.58% vs. 14.23 ± 3.48%, p = 0.001), Rifampicin (78.51 ± 6.82% vs. 67.31 ± 5.73%, *p* = 0.062), and Omeprazole (54.26 ± 4.21% vs. 22.12 ± 2.34%, p = 0.0002). Following induction, the hiPSC-EB-HLCs treated with the two inhibitors displayed similar CYP activity relative to primary hepatocytes for CYP3A4 (78 vs. 82, p = 0.417), but statistically significantly lower CYP activities for CYP2B6 and CYP1A2 relative to primary hepatocytes (28 vs. 98, p < 0.0001, and 54 vs. 98, p = 0.0007, respectively). In comparison, hiPSC-EB-HLCs treated without the two inhibitors had all statistically significantly lower CYP activities for all the isoforms when compared with primary hepatocytes (CYP3A4: 67 vs. 82, p = 0.0232; CYP2B6: 14 vs. 98, p < 0.0001; CYP1A2: 22 vs. 98, p < 0.0001). Undifferentiated hiPSC-EBs did not demonstrate any activities of any of the tested isoforms of CYP450.

### Transplantation of hiPSC-EB-HLCs resulted in prolonged survival and human albumin release

**Figure 6:**
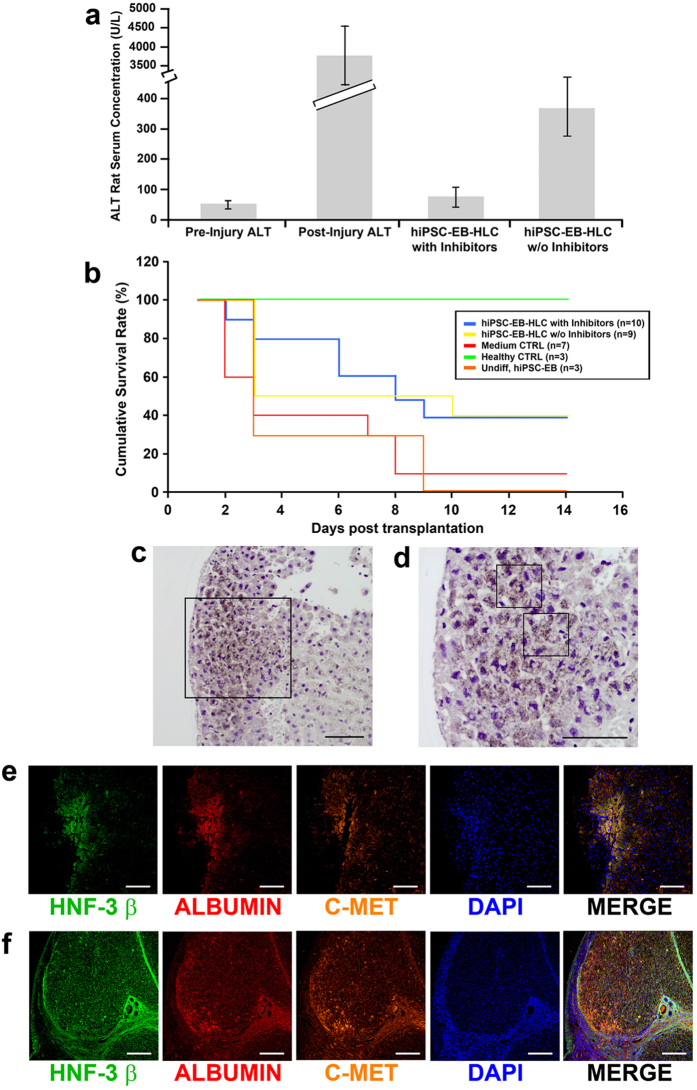
*In vivo* functionality of the hiPSC-EB-HLCs in a rat model of acute liver failure induced by D-Galactosamine. (**a**) Serum level of alanine aminotransferase (ALT). The mean values of ALT prior to liver injury was 53 U/L; after injury was significantly higher at 3781 U/L; and at 2 weeks was 78 U/L following transplantation of hiPSC-EB-HLCs treated with the two inhibitors, and 364 U/L for the hiPSC-EB-HLCs without inhibitors; (**b**) The Kaplan-Meier survivals were determined for 12 days after cell transplantation; (**c**) Histological examination of the liver sections of the survived animals at 14 days after hiPSC-EB-HLCs transplantation showed intense positive staining for human albumin; 20x and 40X. **(e**) Immunofluorescence of the rat liver after transplantation showed the co-staining of several human hepatic proteins such as HNF-3β, Albumin and C-MET; (**f**) Immunofluorescence of human liver was used as positive control displaying staining of all three human hepatic proteins.

The d-galactosamine-induced model of acute liver failure in rats resulted in widespread hepatic necrosis within 24 to 48 hours after injury. Deaths occurred as early as 2 to 3 days after induction of liver failure and nearly 100% mortality was reached within 9 to 10 days after induction. Cell transplantation was performed 14 to 16 hours after induction of liver injury. Alanine aminotransferase (ALT) was used as a marker of liver injury. The mean ALT value (3781 U/L) increased significantly relative to the pre-injury condition (53 U/L), which then normalized to 78 U/L following transplantation of hiPSC-EB-HLCs treated with the inhibitors and 364 U/L for the hiPSC-EB-HLCs without inhibitors, indicating resolution of lethal liver injury for both experimental conditions (Fig. 6a).

The Kaplan-Meier survivals were determined for 14 days after cell transplantation. Almost all the no-cell medium control animals and the animals receiving undifferentiated hiPSC-EBs died within 5 to 8 days after the induction of liver failure (Fig. 6b). Through the examination period (14 days), animals receiving the hiPSC-EB-HLCs treated with the inhibitors trended towards higher mean survival (Fig. 6b) compared to the ones receiving the hiPSC-EB-HLCs without inhibitors (9.0 ± 4.76 vs. 8.33 ± 5.98 p = 0.7902) (Table 1).

Examination of human albumin in the rat’s serum after cell transplantation indicated persistent secretion of human albumin in the animals receiving hiPSC-EB-HLCs with or without inhibitors. In particular, at 72 hours after transplantation both groups of rats that received the clusters with or without inhibitors displayed human albumin in their serum at a concentration of 1.63 ± 0.43 ng/mL and 0.20 ± 0.05 ng/mL respectively. At 14 days after cell transplantation, the concentration of human albumin in the rats serum increased in both experimental groups with and without inhibitors reaching the values of 28.20 ± 7.8 ng/mL and 18.80 ± 5.4 ng/mL respectively. The overall results for both experimental groups showed that human albumin was detected in nearly 80% of the survived animals receiving the hiPSC-EB-HLCs treated with WIF-1 and DKK-1 and 66% in the rats transplanted with the hiPSC-EB-HLCs differentiated without the two inhibitors. None of the control groups at any time showed human albumin in their serum (Table 1).

Figure 6c,d show representative patterns of positive staining of human albumin in the livers of the hiPSC-EB-HLC transplantation group at 14 days post-transplantation. Spleen sections in all animals in this group were negative for human albumin staining. Co-expressions of all three human hepatic proteins (HNF-3 β, human albumin, and C-MET) by the transplanted hiPSC-EB-HLCs in these rat livers were seen throughout the examination period of 14 days post-transplantation using the immunohistochemical staining of the whole liver (Fig. 6e). The staining specificity was confirmed using human liver as a positive control (Fig. 6f).

## Discussion

Improved understanding of the events and the stage-specific inducing factors that are implicated in physiological hepatogenesis has contributed to the development of differentiation culture protocols to derive HLCs from hiPSCs *in vitro*. In general, existing protocols to differentiate hESCs or hiPSCs into HLCs are limited by two issues: low differentiation efficiency and high heterogeneity of the resultant cell populations. In one study, hESCs plated on adherence culture on MEF feeder layers underwent 2-step differentiation, first into definitive endoderm, then to hepatocytes on a collagen I matrix in serum-free medium through stepwise addition of inducing factors that were involved in early and late hepatic development^54^. The differentiating cultures exhibited sequential expression of stage-specific hepatic genes, a hepatic differentiation yield of nearly 70%, *in vitro* functional hepatocyte characteristics, and repopulation of the remnant liver in a mouse model of liver injury. During the differentiation process, the differentiated cells demonstrated progressive loss of expression of the pluripotent markers Oct4 while gaining strong expression of early-stages hepatic proteins Sox17, FoxA2, and Gata4, followed by late-stage hepatic proteins albumin, CD26, and AAT, consistent with increased specification toward hepatic lineage. Despite these findings, the *in vitro* adherence culture-based hESC-derived HLCs retained the expression of immature markers for fetal hepatocytes and exhibited some functional deficiency (e.g., low P450 activities), suggesting incomplete differentiation or cell maturation under the described conditions. Similar to this study, there are other reports on the limitations of existing protocols^16^,^44^,^47^,^54^. To date, differentiation of hiPSCs to cells equivalent to primary hepatocytes has not been achieved.

Conventional hepatic differentiation based upon 2D adherence culture has generated cell populations that differ from primary hepatocytes^16^,^44^,^47^,^54^. 2D differentiation on planar substrates fails to capture the intricate structure of the 3D extracellular environment in native tissue, and therefore constrains the ability to generate cells of phenotypes and properties that closely mimic primary cells *in vivo*. During liver organogenesis, the liver bud is a 3D structure with dynamic cell-cell interactions among multiple cell types during development^66^,^67^,^68^,^69^,^70^,^71^. Cell-cell interactions, particularly through E-cadherin positively impact hepatocyte maturation^72^,^73^. Previous studies have shown that primary hepatocytes and hiPSC-derived HLCs grown in 3D culture retain their hepatic features better when compared to their counterparts in 2D culture^74^,^75^,^76^,^77^,^78^,^79^,^80^,^81^. Most of the published protocols for the hepatic differentiation of hiPSCs or hESCs into HLCs have paired 2D culture during early stage of differentiation with subsequent 3D culture to promote assembly of differentiated cells for final maturation^32^,^78^,^82^,^83^,^84^,^85^,^86^. Our differentiation protocol was performed completely in 3D culture, using a new ROCKi-free and spin-free technique for EB formation^29^,^33^. When compared to 2D adherence culture-based differentiation, 3D culture-based differentiation using hiPSC-EBs offers several advantages including greater capacity for high cell density by obviating the cell-cell contact inhibition and growth surface area restrictions in 2D, and promoting maturation of HLCs by cell-cell contact^87^. In addition, differentiated cells in the form of clusters do not require enzymatic or mechanical dissociation before use, thus reducing potential cell damage/loss due to further processing. Clusters of differentiated cells generated in 3D culture are clearly visible, easy to transport, and readily injectable. Our differentiated hiPSC-EB-HLCs in the form of clusters did not demonstrate any core necrosis up to 1,000 μm in diameter, suggesting that the permeability level of the clusters was sufficient to allow oxygen/nutrient exchange and diffusion^85^,^88^,^89^.

Despite the sequential administration of inducing growth factors involved in physiological hepatogenesis to drive the differentiation of hiPSCs through different stages, none of the previously published hepatic differentiation protocols address the inhibition of the Wnt pathway that occurs during *in vivo* liver organogenesis^83^,^90^,^91^,^92^. The effect of Wnt/β-catenin signalling on cell specification toward specific lineages, including hepatocytes, is widely seen during embryogenesis across species^35^. During early liver development, β-catenin expression is highest at E10-E12, followed by a reduction after E16^38^,^39^,^93^,^94^. In hepatogenesis, Wnt modulation occurs at a late stage of cell differentiation, and in conjunction with β-catenin, is crucial in dictating the differentiation of liver progenitor cells (i.e., hepatoblasts) toward hepatocytes or cholangiocytes. When activated, the Wnt/β-catenin pathway drives hepatoblasts toward cholangiocytes, while when inhibited, it drives hepatoblasts toward hepatocytes. These effects of the Wnt/β-catenin pathway have allowed manipulation at the fate-determining hepato-biliary stage during differentiation to increase the yield in one or the other phenotype. By incorporating the inhibitors of the Wnt/β-catenin pathway into the differentiation protocol, it is possible to offset the balance of fate specification into hepatocytes vs. cholangiocytes, therefore enhancing hepatocyte production^38^,^40^. The Wnt/β-catenin pathway is regulated by two classes of antagonists^95^. One is the secreted frizzled-related protein (sFRP) family (e.g., WIF-1) which blocks Wnt signalling through binding to Wnt proteins^96^, and the other is the Dickkopf (DKK) class (e.g., DKK-1) which blocks Wnt signalling through inhibiting the formation of the Wnt-induced Frizzled-LPR5/6 complex^97^,^98^. Wnt proteins are also grouped into two classes: canonical and noncanonical, based upon their activity in cell lines and *in vivo* assays. In theory, sFRP family inhibits both canonical and noncanonical pathways, whereas DKK class specifically inhibits the canonical pathway^95^. In particular, DKK-1 inhibits Wnt-induced stabilization of β-catenin^99^, and may be specific to the Wnt/β-catenin pathway. In the current work, we administrated inhibitors from both classes, i.e., WIF-1 and DKK-1, in the hope that they may act synergistically in blocking the Wnt/β-catenin pathway.

The stage-specific temporal gene and protein expression profiles of our hiPSC-EB-HLCs are consistent with previous reports^66^,^67^,^68^,^69^,^70^,^71^, confirming a stepwise differentiation into mature hepatocytes using our protocol. Our protocol recapitulates *in vitro* the four stages seen in liver development during normal embryogenesis, starting with the pluripotent state (PS), definitive endoderm (DE), foregut endoderm (FE), hepatic progenitors or hepatoblast (HP), and mature hepatocytes (MH). There was overlap in the gene expression of each stage. Addition of the two inhibitors of the Wnt/β-catenin pathway at a late stage during our hEB-based 3D hepatic differentiation program has increased the commitment of hepatoblasts toward mature hepatocytes while suppressing the production of other cell types, specifically cholangiocytes. There was a significantly higher yield of mature hepatocytes (over 80%) following differentiation in the presence of both Wnt inhibitors (WIF-1 and DKK-1) relative to the differentiated cells in the absence of both Wnt inhibitors highlighted by the presence of human albumin production. The Wnt inhibitors also address the issues of incomplete differentiation and maturation that are associated with conventional protocols. *In vitro*, our hiPSC-EB-HLCs displayed a full spectrum of functionality of mature hepatocytes including albumin secretion, detoxification and metabolism through the P450 enzyme family, AFP secretion, Fibrinogen secretion, and lipid and glycogen storage for both groups with and without WIF-1 and DKK-1. Among them are key functions of mature hepatocytes, such as LDL uptake indicative of fatty acid absorption for lipogenesis, glycogen uptake and storage, triglyceride storage as an energy reservoir, and ICG uptake and subsequent clearance showing the ability to metabolize certain substances. ICG is an organic anionic dye that is exclusively eliminated by the liver^100^,^101^. One of the most important functions of hepatocytes is detoxification and metabolism through the P450 enzyme family. This function is essential *in vivo* and *in vitro* for pharmaceutical screening as it helps to determine drug toxicity and tolerance. In addition to constitutive activity, the hiPSC-EB-HLCs from both experimental groups demonstrated the ability to up-regulate specific P450 family CYP enzyme isoforms in response to specific inducers. In our study, three common and physiologically important P450 isoforms were tested^100^,^101^. These findings strongly suggest that the cells have undergone maturation to a mature hepatocyte phenotype^102^,^103^ and are functional in terms of detoxification and metabolism and response to major external stimuli.

The hiPSC-EB-HLCs that we generated in 3D culture in a scalable manner were capable of rescuing animals from acute liver failure in a rat model. Liver failure causes a physiological severe deficiency in hepatic function, and is associated with significant mortality and morbidity worldwide. The only effective treatment to date is liver cellular and solid organ transplantation^102^,^103^. Shortage of liver donors and a low efficiency of primary hepatocytes cell transplantation therapy represent insurmountable obstacles for treatment. In our study, at 2 weeks post-transplantation, no hiPSC-EB-HLCs (human albumin-positive cells) were seen in the spleen, the original site of injection, yet numerous hiPSC-EB-HLCs were clearly seen in the recipient rat livers. These findings are in line with previous reports of intrasplenically transplanted primary hepatocytes of human or animal origin leaving the spleen for nidation in the liver chords^6^, suggesting the replication of a key feature of primary hepatocytes by the hiPSC-EB-HLCs. The transplanted hiPSC-EB-HLCs persistently secreted human albumin into the host plasma throughout the examination period (72 hours and 14 days), and successfully bridged the animals subjected to acute liver failure through the critical period for survival, providing a promising clue of integration and full *in vivo* functionality of these cells. In particular, the animals transplanted with hiPSC-EB-HLCs treated with the two inhibitors displayed a higher concentration of human albumin in their serum compared with the ones that were transplanted with hiPSC-EB-HLCs without inhibitors.

All our experiments were carried out comparing the hiPSC-EB-HLCs differentiated using two protocols with and without WIF-1 and DKK-1. The comparison between the two conditions showed that when WIF-1 and DKK-1 were added, the differentiation process was enhanced as demonstrated by improved hepatic functionality of the resultant hiPSC-EB-HLCs.

Taken together, our stepwise 3D spheroid culture-based hepatic differentiation protocol involving two inhibitors of the Wnt/β-catenin pathway at a late stage during differentiation has resulted in hiPSC-EB-HLCs that not only bear the genetic and proteomic signatures of adult primary human hepatocytes, but also mature hepatocyte-like functionality both *in vitro* and *in vivo*. The differentiation program is readily scalable and highly efficient. The resultant cell population is homogeneous, fully differentiated, and matured. These cells may provide viable substitutes for primary human hepatocytes in regenerative medicine and pathophysiological studies, as well as pharmacological screening and drug discovery.

## Materials and Methods

### Cell sources and culture conditions

Human induced pluripotent stem cells (hiPSCs) were a foreskin fibroblast-derived cell line iPS(foreskin)-3 (purchased from WiCell Research Institute, Madison, WI– cat# WB0002) and cultured in chemically defined stem cell medium (mTeSR1 basal medium with mTeSR1 supplement, Stem Cell Technologies, Ontario, Canada) on a Matrigel matrix (BD Biosciences, San Jose, CA). iPSC colonies were passaged using Versene (EDTA) (Lonza, Allendale, NJ) for 8 minutes at room temperature.s

### Embryoid body (EB) formation

Agarose micro-well arrays were made using locally developed Teflon stamps and low melting point agarose (Sigma-Aldrich). The agarose, 40 g L^−1^, was dissolved in phosphate buffered saline (PBS) at 100 °C and pipetted into the culture ware. The Teflon stamps were pressed into the agarose solution for approximately 5 minutes. The agarose gelled in about 2 minutes and the stamp was withdrawn with resultant microwell arrays in the agarose gel substrate. After the agarose gelled, arrays were primed by incubation with EB differentiation medium (1:1 mixture IMDM and F-12 Nutrient Mixture (Ham) (Invitrogen), 5% fetal bovine serum (Invitrogen), 1% (vol/vol) insulin transferrin selenium-A supplement (Invitrogen), 55 μM monothioglycerol (Sigma-Aldrich), 100 U L^−1^ penicillin, and 0.1 mg L^−1^ streptomycin (Invitrogen) overnight at 37 °C and 5% CO_2_.

For hiPSC-EBs formation, 1.2 × 10^6^ dissociated hiPSC in a 50 μl suspension were placed in each microwell array and allowed to sediment into the microwells. After 24-hour incubation at 37 °C, three-dimensional EB were aspirated from the microwells and transferred to a 35 mm tissue culture dish (BD Biosciences). The cells were kept in suspension culture in basal hepatocyte medium under gentle agitation on an orbital shaker at 37 °C and 5% CO_2_ with medium changes every other day.

### Hepatic differentiation procedure

Our four-stage *in vitro* hepatic differentiation protocol sought to recapitulate the changes that occur during embryogenesis. The four stages are definitive endoderm, foregut endoderm, hepatobiliary progenitor and committed hepatocyte. Each stage of the differentiation protocol lasted four days with two every-other-day medium changes and addition of the soluble differentiation factors.

The basal differentiation medium consisted of IMDM with F-12 Nutrient Mixture (Ham), 5% fetal bovine serum, 1% (vol/vol) insulin transferrin selenium-A supplement, 55 μM monothioglycerol, 100 U ml^−1^ penicillin, and 0.1 mg ml^−1^ streptomycin (Sigma-Aldrich). Differentiation towards the definitive endodermal stage was promoted through the addition of 10 ng ml^−1^ basic FGF, 100 ng ml^−1^ Activin-A and 10 ng ml^−1^ TGF-β (all from PeproTech, Rocky Hill, NJ). The second foregut endoderm stage was promoted through the addition of 10 ng ml^−1^ FGF-4 (PeproTech) and 10 ng ml^−1^ BMP-4 (Invitrogen). Following endodermal commitment, absence of Wnt signaling is integral to hepatobiliary differentiation. The addition of Wnt pathway inhibitors, 1 μg ml^−1^ WIF-1 (R&D System, Minneapolis, MN) and 0.1 μg ml^−1^ DKK-1 (PeproTech), served to suppress Wnt signaling and promote the third stage of differentiation. Following hepatobiliary commitment, the presence of HGF and oncostatin determines differentiation into cholangiocytes or hepatocytes. By adding 50 ng ml^−1^ HGF (PeproTech) and 30 ng ml^−1^ Oncostatin A (PeproTech), we directed the hepatobiliary cells into a hepatocyte pathway at the fourth stage.

For the embryoid body differentiation, all factors were added to the cell culture media and the embryoid bodies were maintained in suspension through gentle orbital agitation. Before the application of the differentiation protocol, undifferentiated hEBs were collected from the same batch to be used as negative control. All the experiments for the hepatic differentiation with and without inhibitors were performed starting from the same batch of hiPSC-EBs; therefore, the samples were analyzed all at the same time at the end of the differentiation process to ensure the reproducibility of our results.

### hEB viability

At the end of the differentiation process, cell viability was evaluated by LIVE/DEAD staining (Catalog # L-7013, Molecular Probes) to determine the presence of any core necrosis according to the manufacturer’s instruction. Fluorescent images were acquired with confocal microscopy using Olympus IX81.

### Gene expression assay

Reverse transcriptase-PCR (RT-PCR) was performed to verify the presence of characteristic gene markers of differentiation. RNA was extracted using Trizol reagent (Invitrogen) and quantified by spectrophotometry (NanoDrop 2000, Thermo-Scientific). RNA was reverse transcribed to cDNA using the MMLV enzyme (Maloney Murine Leukemia Virus Reverse Transcriptase, Promega, Madison, WI). cDNA was amplified using Taq polymerase with the following parameters: one cycle of 94 °C for 4 min, 30–35 cycles of denaturation at 94 °C for 30 sec, and annealing at 60 °C for 30 sec. The following genes were evaluated: Alpha fetoprotein (AFP), Albumin, Cytokeratin 18, and P450 cytochromes Cyp3a4, Cyp2c9, Cyp3a7, Cyp1b1, Cyp2b6, Cyp1a2, CK-7, HNF-1 β, EpCAM, NCAM, Anion Exchanger 2 (AE2), SALL4 and Cyp3a7. GAPDH was used as the reference housekeeping gene. Values were normalized and reported relative to the glyceraldehyde-3-phosphate (GAPDH) housekeeping gene. Error bars represent the standard deviation of three independent experiments. Data is presented as mean ± SD.

For quantitative RT-PCR (qRT-PCR), extracted RNA was treated with RNase-free DNase (Promega) and reverse-transcribed using an iScript cDNA synthesis kit (Bio-Rad) according to manufacturer instructions. Custom PrimePCR plates (Bio-Rad, 96 well, SYBR plate with 9 unique assays, Catalog #10025217) with lyophilized primers of interest were used with SsoAdvanced Universal SYBR green and run according to the manufacturer instructions. The following amplification conditions were used for a total of 40 cycles: activation for 2 minutes at 95 °C, denaturation for 5 seconds at 95 °C, annealing at 60 °C for 30-second melt curve at 65–95 °C (0.5 °C increments) for 5 sec/step. CFX96 Touch (Bio-Rad) was used for the amplification and data was processed using CFX Manager 3.1 (Bio-Rad). Values were normalized and reported relative to the glyceraldehyde-3-phosphate (GAPDH) housekeeping gene.

### Immunofluorescence assay

Embryoid bodies undergoing differentiation were collected at the end of each stage for immunofluorescence analysis of stage-specific markers. The embryoid bodies were fixed with 4% (wt/vol) paraformaldehyde for 90 minutes, permeabilized with 0.3% (vol/vol) Triton-X 100 in PBS for 1 hour, and blocked with 0.5% (vol/vol) goat serum (Sigma-Aldrich) in PBS for 1 hour. Samples were incubated with the primary antibody at 4 °C for three days. After several washes, the samples were then incubated with the secondary antibody at room temperature for 2 hours. The above incubation times were necessary for complete staining, likely due to the large radius of the EB clusters and increased time for diffusion.

The following human specific primary antibodies were used: rabbit anti SOX17 (Santa Cruz, sc-20099; 1:100); mouse anti FOXA2 (Abcam, ab60721); 5 μg ml^−1^, goat anti Hhex (Santa Cruz, sc-15128; 1:100); mouse anti GATA-4 (Santa Cruz, sc-25310; 1:100); mouse anti AFP (Santa Cruz, sc-166325; 1:100); mouse anti HNF-4α (Santa Cruz, sc-8987; 1:100); goat anti Albumin (Santa Cruz, Santa Cruz, CA, sc-46293; 1:100); mouse anti Cytokeratin 18 (CK-18) (Abcam, ab82254, 5 μg ml^−1^); mouse anti HNF1-α (Santa Cruz, sc-135939; 1:100); and rabbit anti human C-MET (Santa Cruz, sc-10; 1:100). The following secondary antibodies were used: Cy2-AffiniPure goat to mouse IgG; Fc Subclass 1 Specific (Jackson ImmunoResearch, 1:100); Cy3-AffiniPure goat to rabbit IgG (H + L) (Jackson ImmunoResearch, 1:100) and Cy5-conjugated AffiniPure rabbit to goat IgG (Jackson ImmunoResearch, 1:100). Nuclei were counter-stained with 4′6-diamidino-2-phenylindole (DAPI) in PBS for 1 hour. Fluorescent images were acquired with confocal microscopy using Olympus IX81. The yield of albumin-producing cells obtained with our differentiation protocol was determined by counting the number of albumin-positive cells over the total number of cells in each optical cross-section using a confocal microscope, and averaged over a minimum of 10 microscopic fields for each cluster and a minimum of 50 different clusters per differentiation condition.

### FACS analysis and cell sorting

After completion of the differentiation protocol, 100 hiPSC-EB-HLC with and without inhibitors were digested using trypsin for 15 minutes at 37 °C. Live/Dead Yellow Fixable Stain was utilized to assess viability. Intracellular Albumin staining was performed using the Fixation/Permeabilization Staining Buffer Set (eBioscience, San Diego, CA). For the FACS analysis the following monoclonal antibody was used: anti-Human Serum Albumin APC-conjugated Antibody (R & D SYSTEMS). Data acquisition was performed on BD FACS Aria II instrument. Purity after sorting was routinely >95%. We drew the threshold based on the control sample stained for viability (live/dead stain) but not for albumin. A gate was drawn so that the frequency of this control sample was considered the zero. All the events above the threshold in the stained samples were deemed as positive. Analysis was performed using FlowJo software. Mean fluorescence intensities (MFIs) were calculated using the geometric mean of the appropriate fluorescence channel in FlowJo. Expansion Indices were determined using the embedded FlowJo algorithm.

### Albumin, AFP and Fibrinogen secretion assays

After 24 hours of the last change of medium, conditioned medium coming from fully differentiated hEBs was collected and stored at −80 °C. Albumin secreted from the differentiated embryoid bodies into the culture media was quantified using a Human Albumin ELISA kit (Abcam ab108788) according to the manufacturer’s instructions. For the Alpha-fetoprotein secretion assay the quantification was performed using an Alpha Fetoprotein Human SimpleStep ELISA kit (Abcam ab193765) according the manufacturer’s instructions. The Fibrinogen secretion into the culture supernatant was quantified using a Fibrinogen Human SimpleStep ELISA Kit (abcam–ab171578) following the manufacturer’s instructions. All the samples were carried out in triplicate.

### Intracellular Urea content assay

Total Urea content within the differentiated hEBs was performed using the whole clusters that were digested with a specific buffer coming from a commercial Urea Assay Kit (abcam–ab83362) according to the manufacturer’s instructions.

### Indocyanin Green Uptake and Release assay

Fully differentiated hEB were incubated with indocyanin green (IGC, Sigma-Aldrich) in basal medium for 1 hour at 37 °C according to the manufacturer’s instructions. Uptake of IGC was detected with light microscopy using an Olympus IX81. IGC release was detected 6 hours later to ensure that all the positive cells released the IGC.

### Uptake of Low-Density Lipoproteins (LDL) assay

LDL uptake assay was performed after completion of the differentiation protocol using Dil-Ac-LDL following the manufacturer instruction. (Alfa Aesar–J65597). Briefly, the cells were incubated overnight in serum free pre-incubation media containing 0.1% BSA. The next day the differentiated hEBs were incubated for 5 hours at 37 °C with Dil-Ac-LDL 10 μg/mL in pre-incubation media. After the incubation the cells were washed several times with pre-incubation media and fixed with 4% paraformaldehyde for 1 hour. DAPI staining for the nuclei was performed after fixation for 1 hour at RT. Fluorescent images were acquired with confocal microscopy using an Olympus IX81.

### Periodic Acid-Schiff (PAS) Staining

The glycogen storage of differentiated hEBs was evaluated using PAS staining according to the manufacturer instructions (Sigma-Aldrich). Briefly, the clusters were fixed with 4% paraformaldehyde for 1 hour, then oxidized for 5 minutes with Periodic Acid solution and then washed several times. Following the washes, 15 minutes incubation with Shiff Reagent was performed followed by color development with dH_2_O for 5 minutes. Staining was detected with light microscopy using an Olympus IX81.

### Oil Red Staining

After differentiation, the cells were tested for the lipid vesicle storage using Oil Red O staining according to the manufacturer’s protocol (abcam–ab150678). Briefly, the clusters were fixed with 4% paraformaldehyde for 1 hour, and then incubated for 2 minutes with Propylene Glycol followed by a 6 minute incubation with Oil Red O solution. After the staining, 1 minute incubation with 85% Propylene Glycol was performed followed by 2 washes with dH_2_O. Staining was detected with light microscopy using an Olympus IX81.

### CYP Activity Assay

The Cytochrome P450 enzymes activity was performed using the P450-GloTM Assay Kit (Promega, Madison, WI) according to the manufacturer’s instructions. We tested the activity of different P450 enzymes, in particular the CYP2B6 (P450-Glo CYP2B6 – V8321/2 – Promega, Madison, WI), CYP3A4 (P450-Glo CYP3A4 (Luciferin-IPA) – V9001/2 – Promega, Madison, WI), and the CYP1A2 (P450-Glo CYP1A2 Induction/Inhibition – V8421/2 – Promega, Madison, WI) by incubating them with different inducers. For the CYP2B6 activity assay, undifferentiated hiPSC, primary hepatocytes and differentiated HLCs were incubated with basal medium containing 1000 μM Phenobarbital solution (Sigma), or DMSO (0.1%) for 48 hours. For the CYP3A4 activity assay, undifferentiated hiPSC, primary hepatocytes and differentiated HLCs were incubated with basal medium containing 20 μM Rifampicin solution (Sigma), or DMSO (0.1%) for 48 hours. For the CYP1A2 activity assay, undifferentiated hiPSC, primary hepatocytes and differentiated HLCs were incubated with basal medium containing 50 μM Omeprazole solution (Sigma), or DMSO (0.1%) for 48 hours. Measurement of the activity of each enzyme was performed by reading the luminescence using a luminometer (Synergy H1 Hybrid Reader - Biotek) according to the manufacturer’s instructions. All the experiments were performed in triplicate.

### Ammonia metabolism assay

Ammonia metabolism was evaluated by changes in ammonia concentration in the cell culture supernatant over a 24-hour period after addition of ammonium chloride. 1 mM of ammonium chloride standard was added to the culture dishes containing 100 differentiated embryoid bodies in suspension. Supernatant was collected and ammonium concentration was measured at 1-, 6- and 24-hour intervals after ammonium chloride addition using a colorimetric ammonia assay kit (BioVision, Milpitas, CA).

### Cell transplantation

The Institutional Animal Care and Use Committee (IACUC) approved the use of animals for experimentation in this study. Acute liver failure was induced in 270–350 g athymic nude rats (Crl:NIH-*Foxn1*^*rnu*^, Charles River Laboratories, Wilmington, MA) by intraperitoneal injection of 950 mg kg^−1^ of sterile D-galactosamine dissolved in Hanks Balanced Salt Solution (Sigma-Aldrich). Under inhalational anesthesia, 80–100 hiPSC-EB-HLCs were injected into the spleen body through the caudal pole of the spleen. Following injection, the caudal pole was ligated. The experimental groups consisted of animals transplanted with the hiPSC-EB-HLCs treated with inhibitors and hiPSC-EB-HLCs treated without inhibitors. Negative controls consisted of animals that received hepatocyte medium only and animals transplanted with undifferentiated hiPSCs embryoid bodies. Healthy controls consisted of animals without liver injury transplanted with hiPSC-EBs. The animals were monitored daily and received standard chow and water ad libitum. Animal survival was tracked as a primary end point. Animals were sacrificed after 14 days or earlier if they had moribund appearance or greater than 30% body weight loss in accordance with predefined humane care criteria. All experiments were carried out in accordance with the approved IACUC guidelines.

### Serum analysis

The tail vein was phlebotomized prior to transplantation, 48–72 hours after transplantation, and at time of sacrifice. Concentration of serum alanine transaminase (ALT) in whole blood was measured using VetScan 2.0 (Abaxis, Union City, CA). The presence of human albumin in the rat serum was evaluated using a Human Albumin ELISA Quantitation Set that was non-cross reactive with rat albumin (Bethyl Laboratories).

### Histology and immunohistochemistry

Liver and spleen samples were recovered at sacrifice or death and fixed with 10% neutral buffered formalin. The cells were subsequently embedded in paraffin and sectioned with hematoxylin and eosin staining for histologic assessment.

The paraffin-embedded slides were deparaffinized using xylene-substitute and ethanol and immunohistochemistry was performed on rat liver and spleen sections to identify the presence of human albumin. Following deparaffinization, endogenous peroxidase activity was blocked with 4% hydrogen peroxide.

For human albumin detection, non-specific binding was blocked with 2% donkey serum for 60 minutes (Sigma-Aldrich) and the slides were incubated with a non-cross reactive goat antibody to human albumin primary antibody (Bethyl Laboratories; 1:500) for 60 minutes. The secondary antibody used was HRP-conjugated donkey antibody to goat IgG (Santa Cruz; 1:200) for 60 minutes.

For the immunofluorescence staining of the rat liver sections, the slides were fixed with 4% (wt/vol) paraformaldehyde for 30 minutes, permeabilized with 0.3% (vol/vol) Triton-X 100 in PBS for 30 minutes, and blocked with 0.5% (vol/vol) goat serum (Sigma-Aldrich) in PBS for 1 hr RT. Samples were incubated with the primary antibody at 4 °C overnight. After several washes, the samples were then incubated with the secondary antibody at room temperature for 1 hour. The following human specific primary antibodies were used: mouse anti human HNF-3β (RY-7) (Santa Cruz, sc-101060; 1:100) and rabbit anti human C-MET (Santa Cruz, sc-10; 1:100).

### Statistical analysis

Quantitative data are expressed as mean ± standard deviation. Comparisons were made using Fisher’s exact test or Chi-square tests for categorical variables, and Student t tests or analysis of variance for continuous variables. All statistical analyses were performed using JMP 9.0 (Stata Corp LP, College Station, TX).

### Ethical statement

All the experimental protocols using both hiPSCs and animal studies were approved and performed according to the policy of both Virginia Commonwealth University and Beth Israel Deaconess Medical Center. The Animal Research Facility (ARF) assists investigators in their obligation to plan and conduct animal experiments in accord with the highest scientific, humane and ethical principles. This is achieved by development and maintenance of a comprehensive, high quality animal care program, which is AAALAC accredited and complies with all Federal, State and Local laws. All the animal protocols were approved by the IACUC.

## Additional Information

**How to cite this article**: Pettinato, G. *et al*. Scalable Differentiation of Human iPSCs in a Multicellular Spheroid-based 3D Culture into Hepatocyte-like Cells through Direct Wnt/β-catenin Pathway Inhibition. *Sci. Rep.* **6**, 32888; doi: 10.1038/srep32888 (2016).

## Supplementary Material

Supplementary Information

## Figures and Tables

**Table 1:** Up to 14 days post-transplantation, there was a trend towards longer mean survival of animals receiving hiPSC-EB-HLCs treated with inhibitors relative to the hiPSC-EB-HLCs without inhibitors, but did not reach statistical significance (9.0 vs. 8.33 days, p = 0.7902).

	Survival to 14 days	Mean survival	72 hrs Rat serum - Human albumin	14 days Rat serum - Human albumin	Human albumin
hiPSC-EB-HLC with inhibitors (n = 10)	40.0% (4/10)	9.0 ± 4.76	1.63 ± 0.43 ng/ml	28.20 ± 7. 8 ng/ml	80.0% (8/10)
hiPSC-EB-HLC w/o inhibitors (n = 9)	38.6% (4/9)	8.33 ± 5.98	0.20 ± 0.05 ng/ml	18.80 ± 5. 4 ng/ml	66.0% (6/9)
Undifferentiated iPSC EB (n = 3)	33.3% (1/3)	8.7 ± 5.5	0	0	0% (0/3)
Healthy Control (n = 3)	100% (3/3)	14	0	0	0% (0/3)
Negative Control (media only)	14.3% (1/7)	5.4 ± 4.5	0	0	0% (0/7)

At both time points of examination, i.e., 72 hrs and 14 days post-transplantation, human albumin was detected in the serum of the survived animals receiving hiPSC-EB-HLCs treated with the two inhibitors in a greater amount when compared with the ones receiving the hiPSC-EB-HLCs without inhibitors. Human albumin was not detected in the serum of any of the control animals at any time.
