# Supplementary material for: Scalable Differentiation of Human iPSCs in a Multicellular Spheroid-based 3D Culture into Hepatocyte-like Cells through Direct Wnt/β-catenin Pathway Inhibition
Source: Sci Rep. 2016 Sep 12;6:32888. doi: 10.1038/srep32888 (PMC5018737; doi:10.1038/srep32888)

**Supplementary information**

**Scalable Differentiation of Human iPSCs in a Multicellular Spheroid-based 3D Culture into Hepatocyte-like Cells through Direct Wnt/β-catenin Pathway Inhibition**

Giuseppe Pettinato^1,3,4^, Rajesh Ramanathan^2^, Robert A Fisher^3,*^, Martin J Mangino^2^, Ning Zhang^4,*^, Xuejun Wen^1,*^

**Supplementary Figure 1.** Embryoid bodies ROCKi/Spin free were produced using an agarose micro-well arrays and Teflon stamps. An 80% confluent six-well plate containing 1.2x10^6^ dissociated hiPSC produced approximately 280 embryoid bodies. Scale bar 600 μm.


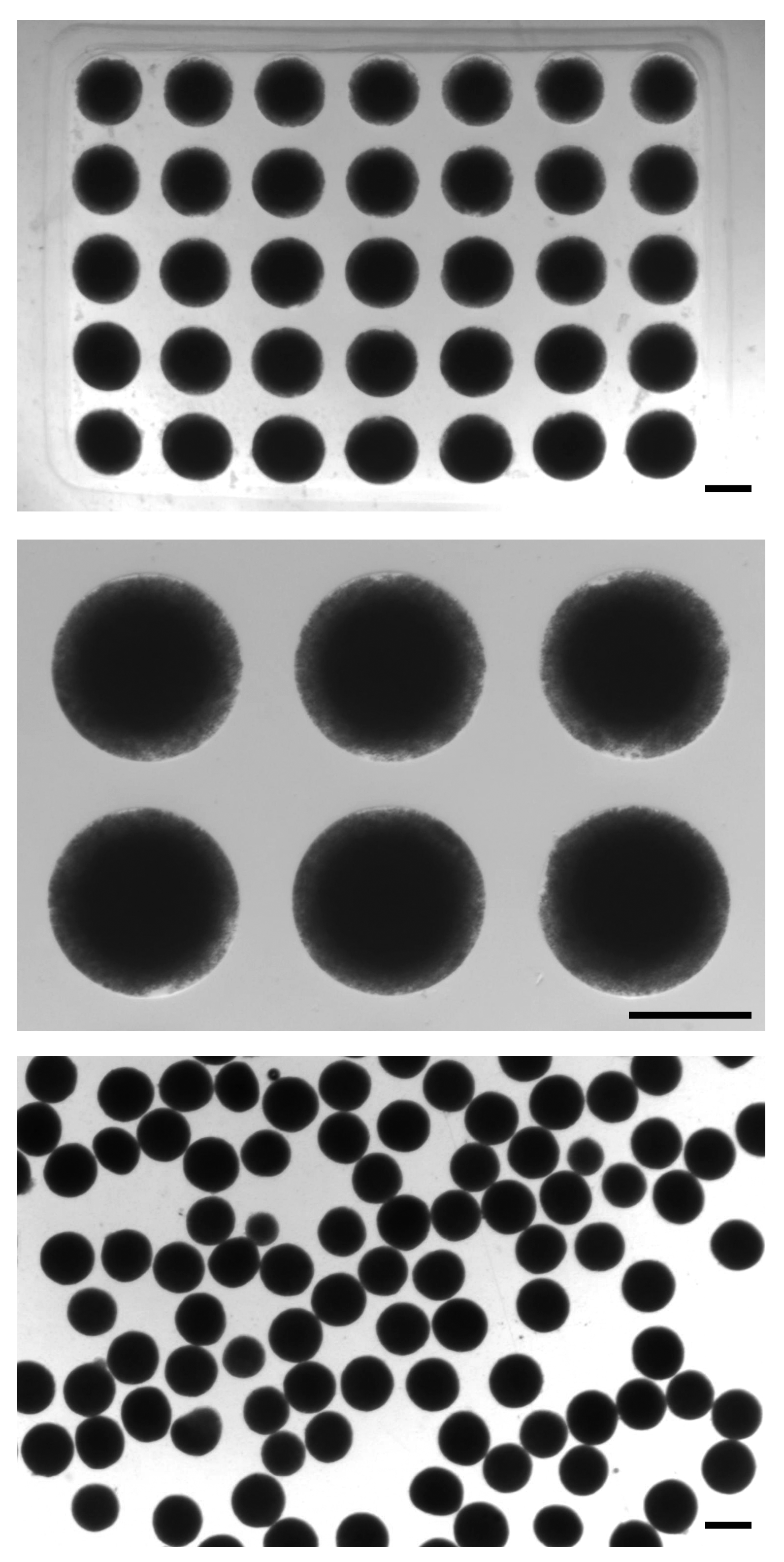


**Supplementary Figure 2.** Representative microscopic fields showing human albumin-producing cells (green) after differentiation. Scale bar 200 μm.


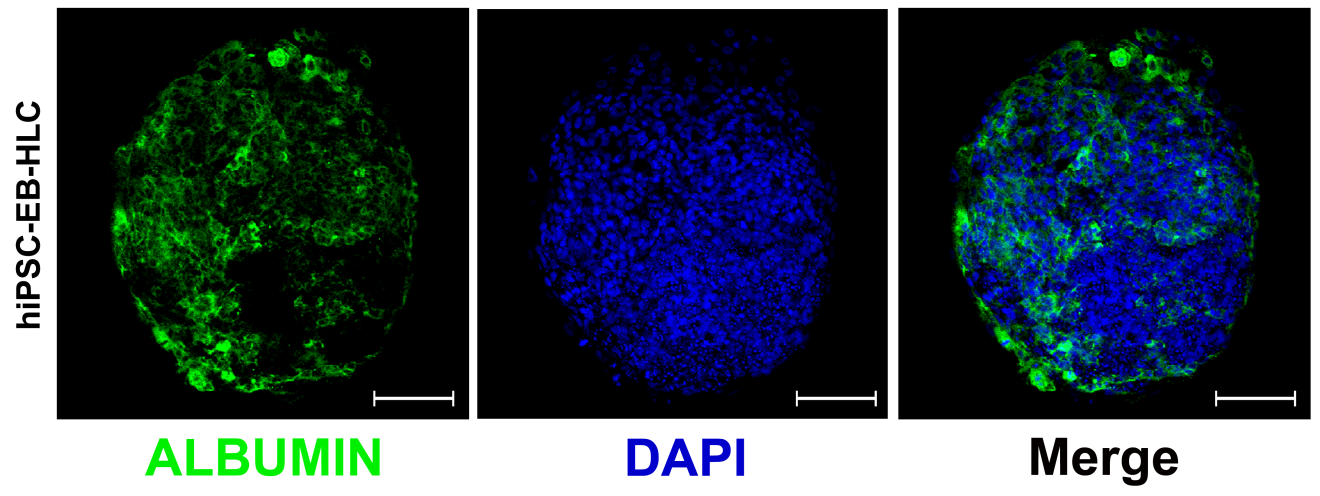


**Supplementary Figure 3.** The hiPSC-EB-HLCs increased in size from approximately 500 µm after 24 hours of their formation to 800-1,000 µm at the end of differentiation process without any core necrosis at any time. The picture shows a live-dead stain of a representative hiPSC-EB-HLC at the end of the differentiation process. Scale bar 200 μm.

**
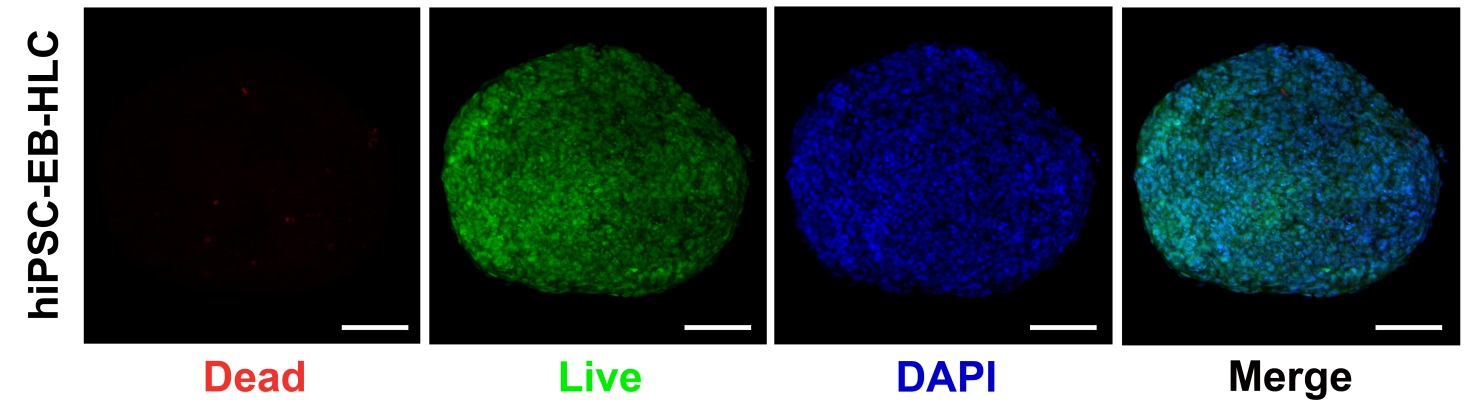
**

**Supplementary Figure 4.** Light microscopy showed that hiPSC-EB-HLCs were morphologically polygonal with enriched cytoplasmic granules (arrows). The differentiated clusters were allowed to attach to a coated plate for morphological examination. Upper picture at 1 week after attachment, lower picture at 2 weeks after attachment. Scale bar 100 μm.

**
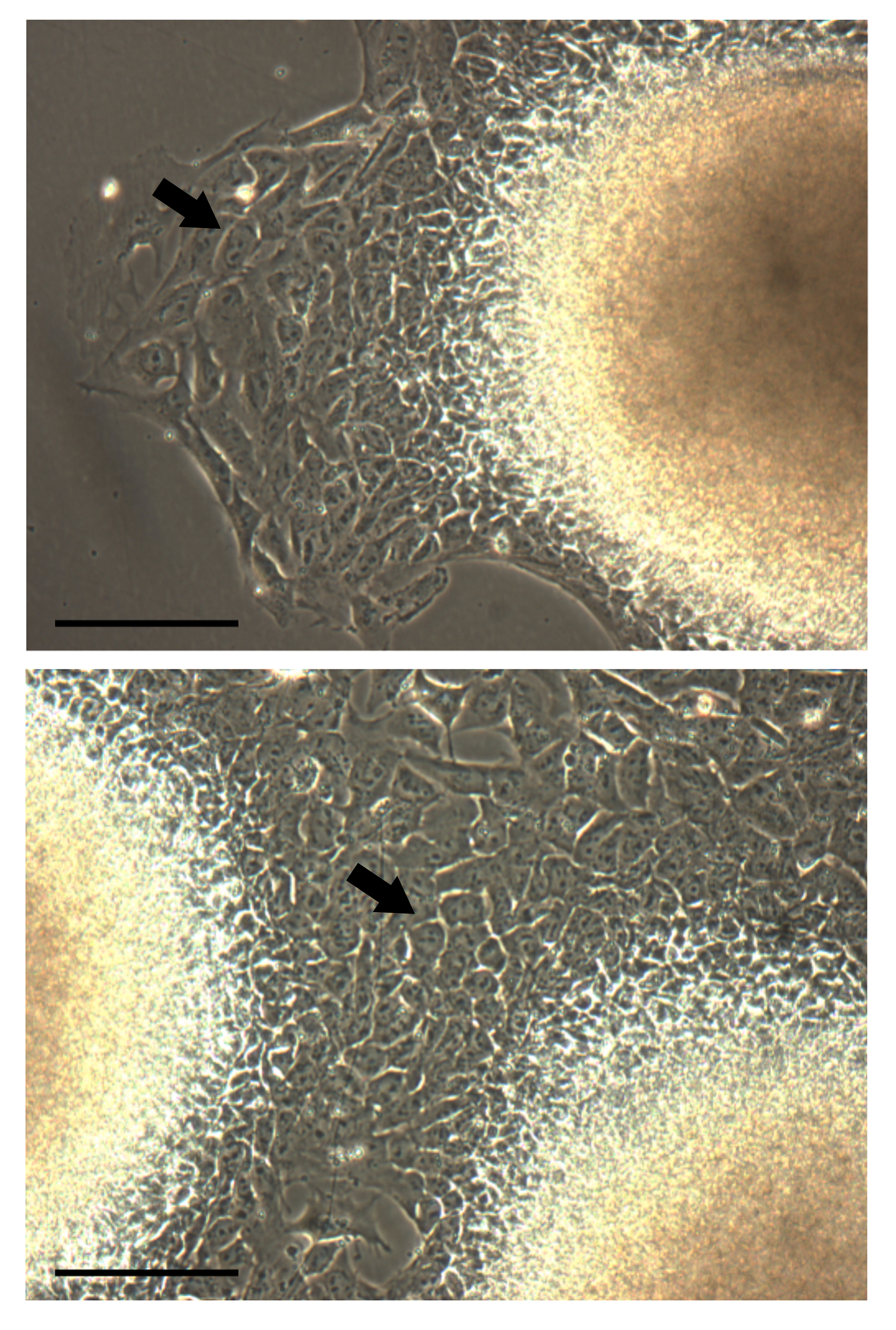
**

**Supplementary Figure 5.** The clusters after spreading onto a matrigel-coated plate, showed a homogeneous distribution of the signal for each functional activity. a) Indocyanin green; b) glycogen storage; c) cytoplasmic accumulation of neutral triglycerides and lipids. Scale bar 200 μm.


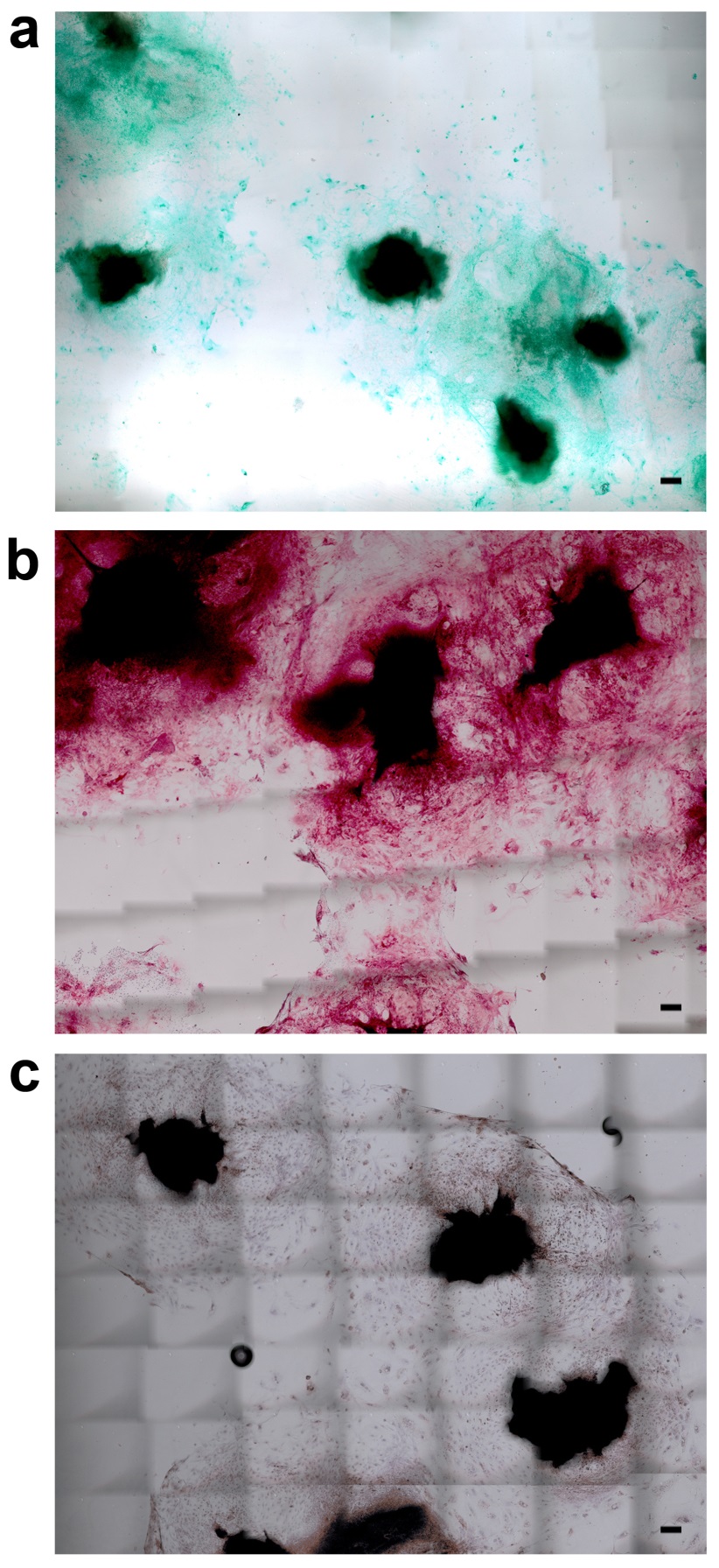

Supplement: Supplementary Information [file srep32888-s1.doc]
